# A Metabolomics Signature Linked To Liver Fibrosis In The Serum Of Transplanted Hepatitis C Patients

**DOI:** 10.1038/s41598-017-10807-y

**Published:** 2017-09-05

**Authors:** Ainara Cano, Zoe Mariño, Oscar Millet, Ibon Martínez-Arranz, Miquel Navasa, Juan Manuel Falcón-Pérez, Miriam Pérez-Cormenzana, Joan Caballería, Nieves Embade, Xavier Forns, Jaume Bosch, Azucena Castro, José María Mato

**Affiliations:** 1 OWL, Parque Tecnológico de Bizkaia, Derio, 48160 Bizkaia Spain; 2 Liver Unit, Hospital Clínic, Centro de Investigación Biomédica en Red de Enfermedades Hepáticas y Digestivas (CIBERehd); Institut d’Investigacions Biomediques August Pi Sunyer (IDIBAPS), Barcelona, Spain; 3 Metabolomic Unit, CIC bioGUNE, CIBERehd, Parque Tecnológico de Bizkaia, Derio, 48160 Spain; 4 Ikerbasque, Basque Foundation for Science, Bilbao, Bizkaia Spain

## Abstract

Liver fibrosis must be evaluated in patients with hepatitis C virus (HCV) after liver transplantation because its severity affects their prognosis and the recurrence of HCV. Since invasive biopsy is still the gold standard to identify patients at risk of graft loss from rapid fibrosis progression, it becomes crucial the development of new accurate, non-invasive methods that allow repetitive examination of the patients. Therefore, we have developed a non-invasive, accurate model to distinguish those patients with different liver fibrosis stages. Two hundred and three patients with HCV were histologically classified (METAVIR) into five categories of fibrosis one year after liver transplantation. In this cross-sectional study, patients at fibrosis stages F0-F1 (n = 134) were categorised as “slow fibrosers” and F2-F4 (n = 69) as “rapid fibrosers”. Chloroform/methanol serum extracts were analysed by reverse ultra-high performance liquid chromatography coupled to mass spectrometry. A diagnostic model was built through linear discriminant analyses. An algorithm consisting of two sphingomyelins and two phosphatidylcholines accurately classifies rapid and slow fibrosers after transplantation. The proposed model yielded an AUROC of 0.92, 71% sensitivity, 85% specificity, and 84% accuracy. Moreover, specific bile acids and sphingomyelins increased notably along with liver fibrosis severity, differentiating between rapid and slow fibrosers.

## Introduction

Liver cirrhosis is the 14^th^ most common cause of death all over the world and the 4^th^ in central Europe. It leads to 1.03 million deaths per year in the world^1^, and 170,000 deaths per year in Europe^2^. Hepatitis C virus (HCV) is the leading cause of cirrhosis and liver transplantation (LT)^3^. Besides, graft infection in viremic patients is universal after liver LT and the most frequent reason for graft loss and reduced patient survival after LT^4^. Nevertheless, the rate of fibrosis progression in transplant recipients is variable and, although approximately 70% present slowly progressive disease, some evolve rapidly to significant fibrosis^5^.

Historically, liver biopsy has been considered to be the gold standard to evaluate most cases of liver disease. Yet, it is well known that this procedure has several limitations, including intraobserver and interobserver variation, the morbidity associated to this invasive technique, and its elevated cost. Besides, sampling variability of liver biopsy may be a problem in individuals with rapid fibrosis progression^6^. All these evidences make crucial the development of new accurate, non-invasive methods that allow repetitive examination of the patients to detect fibrosis in earlier stages in order to adopt therapeutic decisions^7^.

Non-invasive methods rely on two different approaches; those that are either based on the quantification of biomarkers in serum or on the measurement of liver stiffness^7, 8^. Serum markers are becoming increasingly useful in the diagnosis of liver fibrosis. Some of them include AST to Platelet Ratio (APRI), Forns index, MP3, Fibrosis Probability Index (FPI), Lok index, Gotebörg University Cirrhosis Index (GUCI), Virahep-C model Fibroindex, FIB-4, HALT-C, Enhanced Liver Fibrosis score^®^ (ELF), Fibrotest^®^, Hepascore^®^, Fibrometer^®^, and FIBROSpect II^®^. All these tests are valuable in the exclusion of advanced fibrosis but, together with liver biopsy, do not distinguish well early and intermediate stages of fibrosis^9, 10^. As revised^11, 12^, classical imaging procedures ultrasonography (U/S), computed tomography (CT), and magnetic resonance imaging (MRI) are used in clinical practice for the detection of advanced liver disease. Transient elastography (TE), Fibroscan^®^, is probably the most widely used non-invasive method in Europe, however, it has limited applicability in case of obesity or under limited operator experience. Recent techniques such as 3-D magnetic resonance (MR) elastography are currently used only for research purposes since they are too costly and time-consuming^13–15^.

HCV infection induces liver fibrosis by complex and not well-understood molecular mechanisms^16^. When HCV infects the liver, it increases lipogenesis and decreases lipid degradation by stimulating lipid droplet synthesis and diminishing very low density lipoprotein (VLDL) assembly and secretion into the blood stream at an advanced stage of infection^17^. The synthesis of lipoproteins is one of the key functions of the liver, therefore, several liver alterations are mirrored in the serum metabolome and constitute potential drug targets and diagnostic clues for liver fibrosis. An elegant study from Kotronen *et al*. demonstrated that the composition of most plasma lipids and the activity of a number of enzymes are correlated in the liver^18^. Serum metabolic profiling strategies are being carried out to learn more about the biochemical pathways involved in disease^19–22^. Moreover, they are aimed to diagnose any pathological state or, even detect its presence before it appears^23^. In fact, plasma lipid profiling has already been applied to understand liver pathophysiology, and to identify biomarkers for fibrosis in mice^24^, hepatotoxicity^25^, hepatocellular carcinoma (HCC)^26–28^, non-alcoholic fatty liver disease (NAFLD)^21, 29–31^, and idiopathic non-cirrhotic portal hypertension^32, 33^. Even to predict survival in patients with decompensated cirrhosis^34^ and to disclose donor liver biomarkers associated with early allograft dysfunction^35^. Lately, some studies have suggested the potential applicability of metabolomics to investigate organ transplantation^35–37^, although applying metabolomics in LT is still in its beginnings^38–40^.

In this study, a predictive metabolomics signature linked to liver fibrosis severity has been found in patients with HCV after transplantation. Rapid (F2-F4) and slow fibrosers (F0-F1) are accurately classified with an algorithm consisting of four lipid metabolites, two sphingomyelins (SM): SM(d18:2/16:0) and SM(38:1); SM(d18:1/20:0) + SM(16:1/22:0) and two phosphatidylcholines (PC): PC(16:0/16:0), and PC(16:0/18:0). The levels of glycocholic acid (GCA), taurochenodeoxycholic acid (TCDCA), SM(d18:0/18:0), and SM(d18:0/14:0) increased very significantly along with the severity of liver fibrosis and differentiate between rapid and slow fibrosers in the univariate and in the multivariate analyses applied. In contrast, the ratio of branched-chain amino acids (BCAA) to aromatic amino acids (ArAA) decreased in rapid fibrosers. The proposed lipid signature will aid to learn about the molecular mechanisms leading to fibrosis after LT and to generate novel, precise, non-invasive clinical tools.

## Results

### Characteristics of the cohorts

Two hundred and three HCV-infected patients were biopsied and classified into five categories of fibrosis one year after transplantation by the validated semi-quantitative METAVIR fibrosis score: F0 (n = 81), F1 (n = 53), F2 (n = 41), F3 (n = 22), and F4 or cirrhosis (n = 6). Clinical, biochemical, and liver histology data obtained from the study participants are summarized in Table 1. All patients were Caucasian and the average recipient age was higher than donor age (66 and 47, respectively). In general, there were more men recipients and donors, except for cirrhotic patients, which were 33.3%. As expected in the studied geographic area, the most frequent virus genotype was 1b. Overall, 57% patients of the cohort (n = 118) had diabetes and received appropriate treatment at one year after LT. Indeed, this percentage was significantly higher in patients classified as rapid fibrosers (72.5%) when compared with slow fibrosers (50.4%), p = 0.002. Serum from HCV liver recipients was obtained at one year after LT for metabolite measurement and histopathology concordance. The study was approved by the Barcelona Clínic Hospital ethics committee and all patients signed informed written consent.

**Table 1:** Patient and donor clinical data. Data is expressed as mean ± standard deviation or in percentage. HCV: hepatitis C virus. 12 M: 12 months after liver transplantation. Alanine aminotransferase (ALT), gamma-glutamyl transpeptidase (GGT).

	**F0 (n = 81)**	**F1 (n = 53)**	**F2 (n = 41)**	**F3 (n = 22)**	**F4 (n = 6)**
Age (years)	64.8 ± 8.5	65.6 ± 7.5	64.2 ± 9.1	68.4 ± 7.3	67.5 ± 8.9
Donor age	42.7 ± 16.7	46.9 ± 18.2	50.3 ± 18.5	45.9 ± 17.5	48.8 ± 15.5
Male (%)	67.9	60	68.3	54.5	33.3
Donor: male (%)	58.8	72	68.3	81.8	83.3
Aetiology (cases HCV + alcohol)	20	13	10	5	1
Hepatocellular carcinoma (No.)	47	27	22	15	4
Ischemia time (min.)	385	342	399	285	259
Genotype HCV 1a:1b:2:3:4 (%)	9:84:1:4:1	10:81:0:8:0	8:79:3:8:3	5:90:0:5:0	0:100:0:0
Allograft rejection (No.)	18	18	11	4	0
ALT 12 M (U/l)	113 ± 120	144 ± 194	148 ± 111	173 ± 132	183 ± 68
GGT 12 M (U/l)	234 ± 400	313 ± 518	365 ± 404	651 ± 1013	970 ± 863
Alkaline phosphatase 12 M (mg/dl)	391 ± 407	370 ± 271	466 ± 611	683 ± 590	784 ± 234
Bilirubin 12 M	1.2 ± 1.1	1.1 ± 0.7	1.5 ± 1.1	2.0 ± 1.8	3.2 ± 3.3
Viral load (Log) 12 M	6.2 ± 0.7	6.1 ± 0.7	6.3 ± 0.6	6.0 ± 1.4	6.5 ± 0.3
Diabetes type II 12 M (% cases)	50.6	47.2	73.2	68.2	83.3
Arterial hypertension 12 M (% cases)	39.5	35.8	46.3	50.0	83.3

### Four lipids accurately distinguish between rapid and slow fibrosers

**Figure 1:**
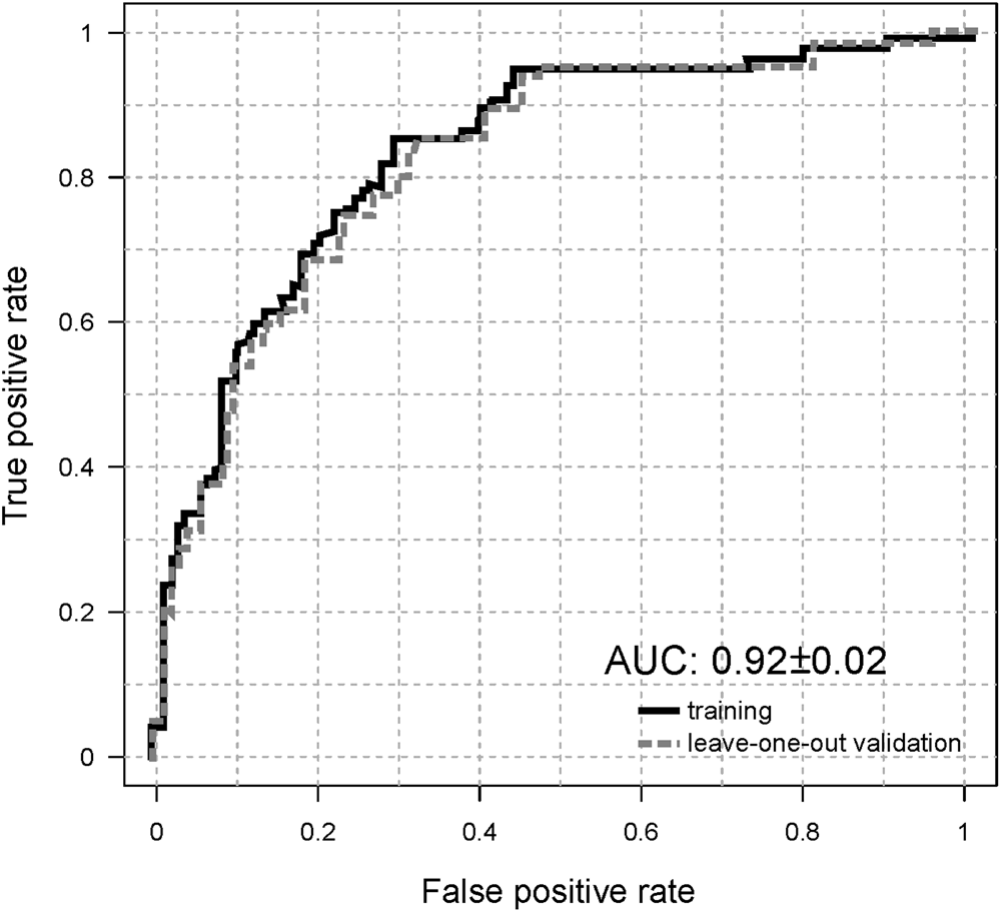
Prediction performance by the area under receiver operating characteristic (AUROC) curve analysis. Metabolic discrimination between rapid (F2-F4) and slow (F0-F1) fibrosers one year after liver transplantation has yielded an AUROC of 0.92, sensitivity 71%, specificity 85%, accuracy 84%, negative predictive value 67%, positive predictive value 84%, positive likelihood ratio 4.73, and negative likelihood ratio 0.34. Linear discriminant analysis has been carried out through LOOCV. Associated ROC curves for the estimation (solid line), and validation (dotted line) data sets. The optimum cut-off point for the estimation group (0.54) was defined as that at which average diagnostic accuracy was a maximum.

A total of 444 metabolites were identified and semi-quantified in the serum of the studied patients. These compounds included 22 amino acids (AA), 45 non-esterified fatty acids (NEFA), 19 oxidised fatty acids, 11 acylcarnitines, 12 diacylglycerols, 69 triacylglycerols (TAG), 14 cholesteryl esters (ChoE), 12 bile acids (BA), 203 glycerophospholipids, and 37 sphingolipids. Among them, four lipid species were combined in an algorithm that differentiates between rapid and slow fibrosers. Based on this model, rapid and slow fibrosers were accurately classified with two SM: SM(d18:2/16:0), and SM(38:1): SM(d18:1/20:0) + SM(16:1/22:0), and two PC: PC(16:0/16:0), and PC(16:0/18:0). This classification model yielded an AUROC of 0.92, 71% sensitivity, 85% specificity, 84% accuracy, 67% negative predictive value, 84% positive predictive value, 4.73 positive likelihood ratio, and 0.34 negative likelihood ratio (Fig. 1). Individual fold-changes and t-test p-values of these four lipid species are shown in Table 2.

**Table 2:** Fold-changes and p-values of the lipid species combined in an algorithm that differentiates between rapid and slow fibrosers (first four compounds); and of the most significant metabolites discriminating between rapid and slow fibrosers by unpaired Student’s t-test (or by Welch’s t test where unequal variances). Data is expressed as mean ± standard deviation. Sphingomyelin (SM), phosphatidylcholine (PC), taurochenodeoxycholic acid (TCDCA), taurocholic acid (TCA), cholesteryl ester (ChoE), glycocholic acid (GCA).

	**Fast**	**Slow**	**Fold-change**	**log**_**2**_ **(fold-change)**	**Student’s t-test (p)**
SM(38:1)	0.6 ± 0.2	0.7 ± 0.2	0.9	-0.2	3.89E-03
SM(d18:2/16:0)	0.8 ± 0.2	0.9 ± 0.2	0.9	-0.1	5.07E-02
PC(16:0/18:0)	0.8 ± 0.2	0.8 ± 0.1	1.0	0.0	7.64E-01
PC(16:0/16:0)	1.3 ± 0.3	1.0 ± 0.2	1.2	0.3	2.92E-08
TCDCA	2.7 ± 1.3	1.3 ± 0.9	2.1	1.1	2.92E-12
TCA	3.0 ± 1.8	1.3 ± 1.2	2.4	1.3	3.71E-11
ChoE(18:1)	0.5 ± 0.2	0.6 ± 0.2	0.8	-0.4	2.88E-08
PC(16:0/16:0)	1.3 ± 0.3	1.0 ± 0.2	1.2	0.3	2.92E-08
Tyrosine	0.7 ± 0.1	0.6 ± 0.1	1.2	0.2	4.94E-07
GCA	1.6 ± 0.8	1.0 ± 0.7	1.6	0.6	6.39E-07
SM(d18:0/18:0)	1.1 ± 0.4	0.9 ± 0.3	1.3	0.3	3.96E-05
PC(32:1)	1.4 ± 0.8	1.0 ± 0.4	1.5	0.6	3.97E-05
PC(O-20:0/0:0)	0.3 ± 0.1	0.3 ± 0.1	0.8	-0.3	4.54E-05
PC(20:0/0:0)	0.4 ± 0.1	0.5 ± 0.1	0.9	-0.2	4.67E-05
SM(d18:0/14:0)	1.1 ± 0.5	0.9 ± 0.3	1.2	0.3	6.05E-04

### Orthogonal partial least squares discriminant analysis performance

**Figure 2:**
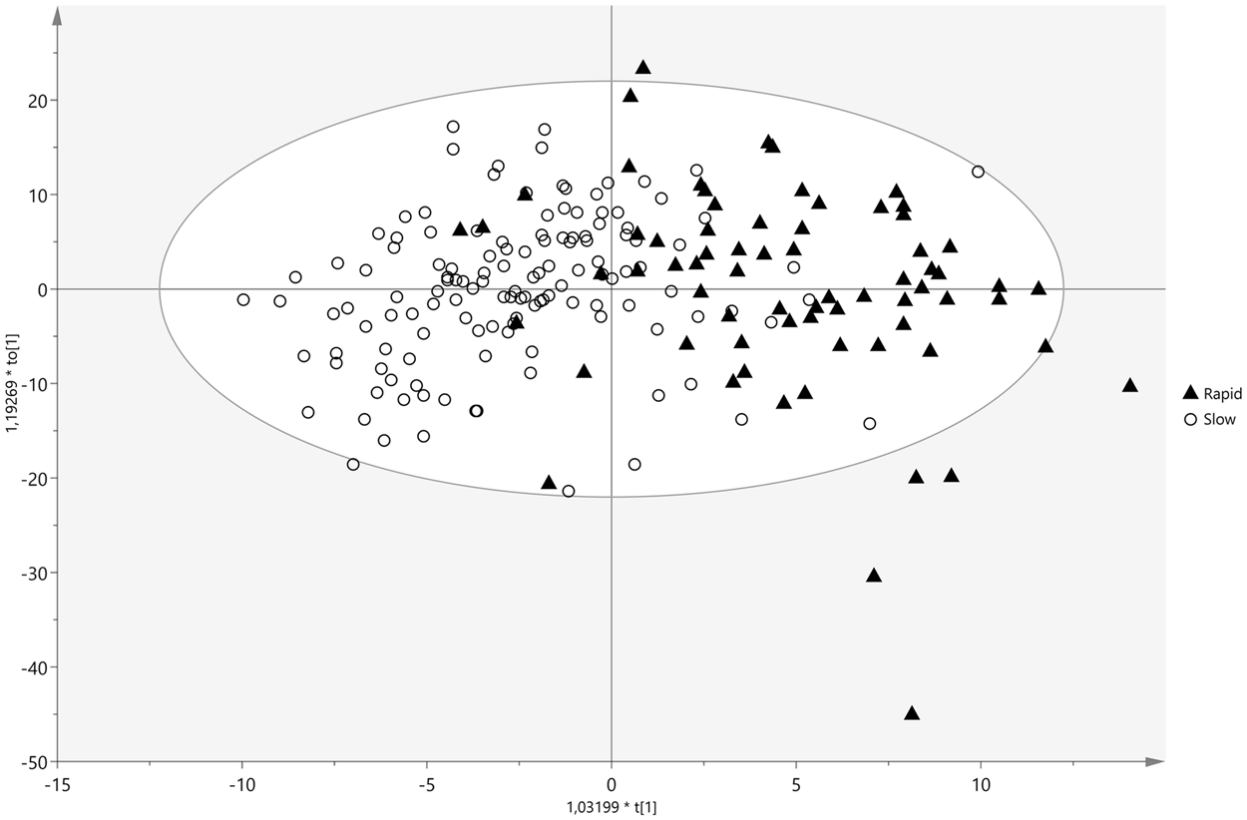
OPLS scores plot of the serum metabolic profiles in patients with hepatitis C virus (HCV). Supervised OPLS scores plot of transplanted patients with rapid (black triangles) and slow (circles) fibrosis progression. Model diagnostics (1 + 2 + 0), R^2^X = 0.32, R^2^Y = 0.502, Q^2^ = 0.27.

**Figure 3:**
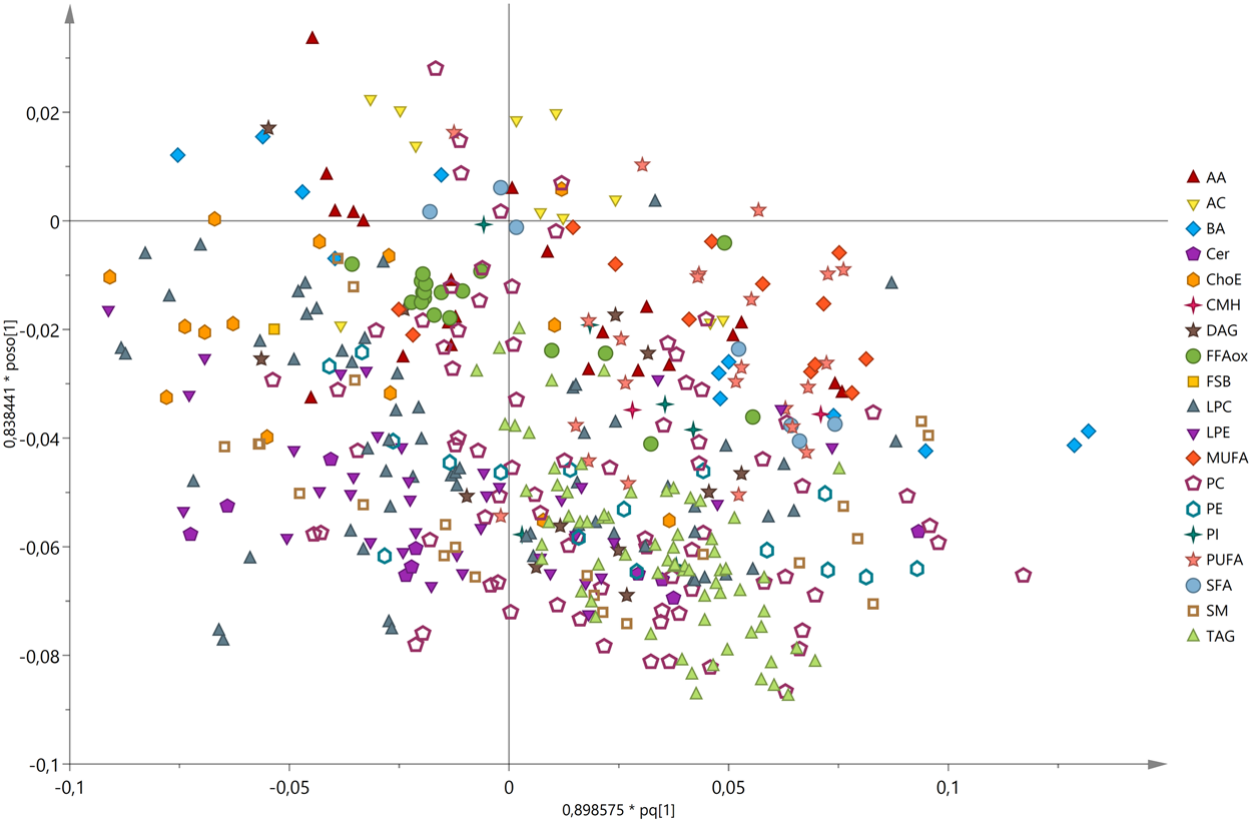
OPLS loadings plot of the serum metabolic profiles in patients with hepatitis C virus (HCV). Supervised OPLS loadings plot of the metabolite species discriminating between rapid (F2-F4; n = 69) and slow (F0-F1; n = 134) fibrosers. Data related to these metabolites is detailed in Supplementary Table S1, sheet “Supp. Table A. OPLS”.

A supervised orthogonal partial least squares discriminant analysis (OPLS-DA) was created in order to identify possible biomarkers discriminating between rapid and slow fibrosers. Two clusters are well differentiated, as patients developing rapid fibrosis lie down on the right hand side of the principal component *t1* while slow fibrosers are positioned on the left hand side (Fig. 2). The performance of the OPLS-DA model was evaluated using the predictive R^2^X and R^2^Y (goodness of fit) and Q^2^Y (goodness of prediction) parameters. This model showed (1 + 2 + 0) components, R^2^X = 0.32, R^2^Y = 0.502, and Q^2^ = 0.27. The loadings plot (Fig. 3) displays the variables responsible for the patterns seen among the samples. Metabolites lying further away from the plot origin have stronger impact on the model; furthermore, variables positively correlated are grouped together, while variables negatively correlated are positioned on opposite sides of the plot origin, in diagonally opposed quadrants.

The metabolites that most discriminate between rapid and slow fibrosers in the multivariate analysis (with the highest loadings absolute values) were mainly BA, PC, lysophosphatidylcholines (LPC), and SM (Supplementary Table S1, sheet “Supp. Table A. OPLS”).

### Metabolomics fingerprint of liver fibrosis

As well as multivariate analyses, univariate studies were performed. The advantages of using both univariate and multivariate approaches in data mining have been recently reviewed^41^. Both approaches are complementary and their results do not necessarily coincide.

**Figure 4:**
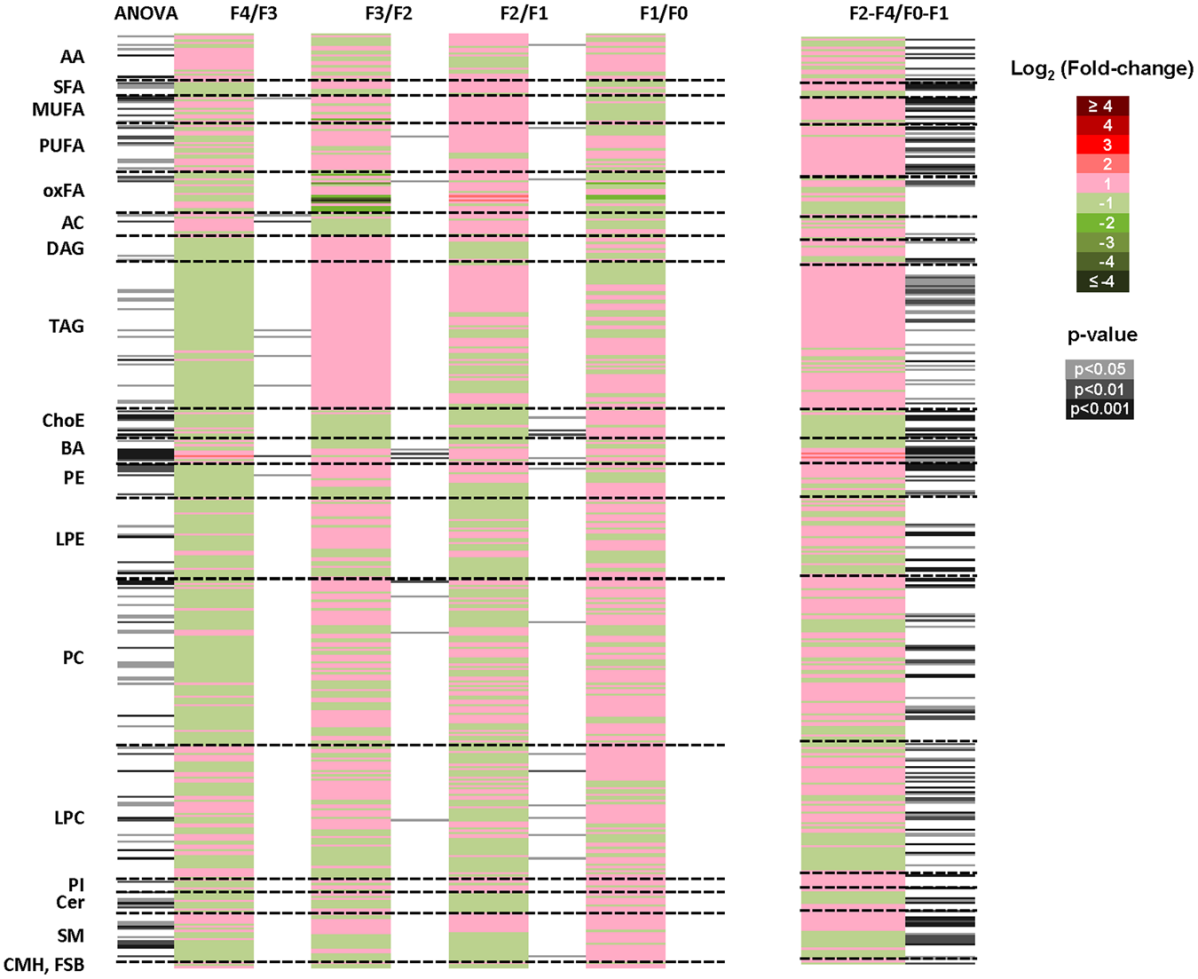
Metabolomics signature in the serum of transplanted patients with HCV. Heatmaps represent fold-changes and p-values derived from the comparison of the relative metabolite levels in serum samples among all fibrosis stage (ANOVA) and between a specific fibrosis degree and its immediate lower fibrosis degree (Tukey’s *post hoc* test) (*i.e*., F4/F3, F3/F2, F2/F1, and F1/F0). Rapid (F2-F4; n = 69) were compared with slow (F0-F1; n = 134) fibrosers with unpaired Student’s t test (or Welch’s t test where unequal variances were found). For each comparison, log transformed ion abundance ratios are depicted, as represented by the scale. Darker green and red colours indicate higher drops or elevations of the metabolite levels, respectively. Grey lines correspond to significant fold-changes of individual metabolite levels, darker grey colours have been used to stress higher significances (p < 0.05, p < 0.01 or p < 0.001). It is relevant to highlight that lipid metabolites present in this picture have been ordered according to the carbon number and unsaturation degree of their acyl chains. Data represented in the figure is detailed in Supplementary Table S1.

Analysis of variance (ANOVA) heatmap displays the fold-changes and p-values for all the fibrosis stages as compared to their immediate lower fibrosis degree (Fig. 4 and Supplementary Table S1, sheet “Supp. Table B. ANOVA”). Despite the reduced number of significant metabolites, this figure underscores a metabolomics signature for each fibrosis degree defined by the fold-changes of the relative metabolite levels in each comparison.

When comparing the levels of metabolites between each fibrosis degree and its immediate previous one by Tukey *post-hoc* analysis, the metabolic profiles of F2 and F1 patients presented the highest number of significant metabolites of all the fibrosis cohorts. The highest significances (p < 10^−3^) were found for LPC(20:0/0:0), ChoE(20:5), and ChoE(22:5), whose levels decreased in F2 patients when compared with F1. Next, TCDCA, taurocholic acid (TCA), and PC(16:0/16:0) were the most significant metabolites in F3 patients when compared with F2. Decanoylcarnitine (10:0) and taurodeoxycholic (TDCA), increased in F4 *vs*. F3 patients. Finally, there were no significant metabolites found in the comparison F1 *vs*. F0 patient groups.

Pearson correlation coefficients (PCC) were calculated to detect the metabolites whose levels changed in line with the stage of liver fibrosis (Supplementary Table S1., sheet “Supp. Table B. ANOVA”). Tyrosine, glutamic acid, threonine, TCDCA, GCA, SM(d18:0/14:0), SM(d18:0/15:0), and SM(d18:0/18:0), nervonic acid, 24:1n-9, 22:3n-x, and LPC(0:0/16:1) increased in parallel with fibrosis stage. In contrast, ceramide (Cer) Cer(d18:1/21:0) and Cer(d18:2/23:0), ursodeoxycholic acid (UDCA), and the ratios BCAA to ArAA, and BCAA to tyrosine (BTR) decreased along with the fibrosis stages of the studied patients (absolute PCC > 0.98).

When comparing the metabolome of rapid and slow fibrosers by unpaired Student’s t-test (or by Welch’s t test where unequal variances were found) metabolites belonging to each chemical group did not display the same fold-change trend (Fig. 4) (i.e., the levels of some PC increased in rapid fibrosers *vs*. slow fibrosers while some others, decreased). The most noteworthy metabolites in this comparison were TCDCA, p = 2.92 10^–12^; TCA, p = 3.71 10^–11^; CGA, p = 6.39 10^–7^; ChoE(18:1), p = 2.88 10^–8^; PC(16:0/16:0), p = 2.92 10^–8^, and amino acid tyrosine, p = 4.94 10^–7^ (Supplementary Table S1, sheet “Supp. Table C. t test”, and volcano; Supp. Figure S1). The levels of these compounds were increased in rapid fibrosers when compared to slow fibrosers except ChoE(18:1), which decreased. A number of metabolites, such as TCA, TCDCA, GCA, ChoE(18:1), PC(16:0/16:0), PC(32:1), PC(20:0/0:0), PC(O-20:0/0:0), SM(d18:0/14:0), and SM(d18:0/18:0) were found also significant in the OPLS-DA. Fold-changes and p-values of these metabolites are summarized in Table 2.

**Figure 5:**
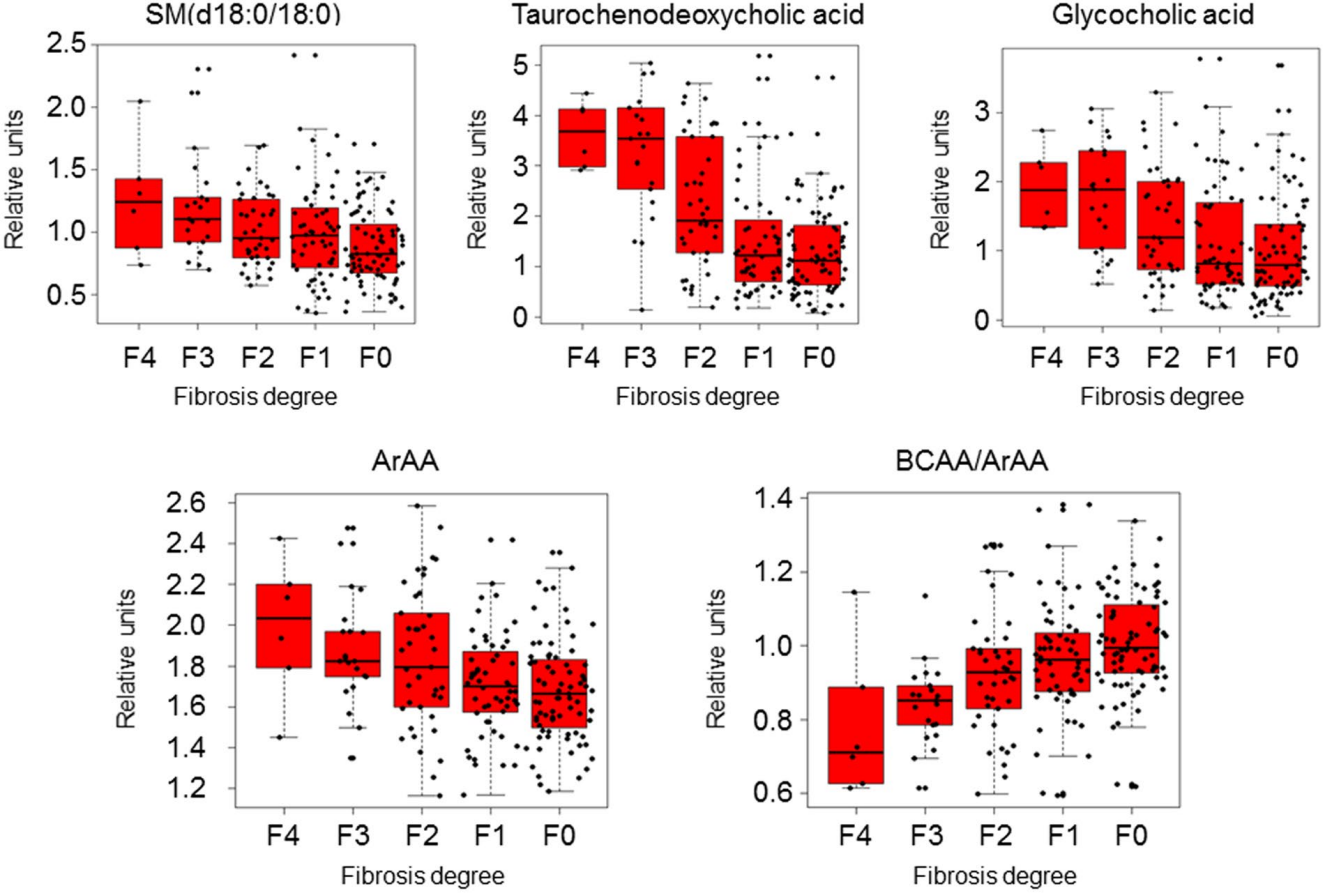
Boxplots of the relative concentrations for the significantly altered metabolites and ratios in serum of patients considering t test, Pearson correlation coefficient and multivariate analyses. Data is shown in the Supplementary Table S1. X axes represent the fibrosis degree (METAVIR Score), Y axes represent relative units of the semiquantified metabolites or ratios. The bar plots show the normalized values. The boxes range from the 25% and the 75% percentiles; the 5% and 95% percentiles are indicated as error bars; single data points are indicated by dots. Medians are indicated by horizontal lines within each box.

In summary, TCDCA, GCA, and SM(d18:0/18:0), which rose very significantly along with the severity of liver fibrosis, were the metabolites that better discriminate between rapid and slow fibrosers in both, univariate and multivariate analyses (Fig. 5, box plots).

## Discussion

We have found a serum metabolomics signature linked to liver fibrosis one year after liver transplantation. In this cross-sectional study, a total of 444 metabolites were semi-quantified in HCV patients with different stages of fibrosis. Among them, only four lipid species, two sphingomyelins and two phosphatidylcholines, were combined in an algorithm that differentiates between rapid and slow fibrosers. This classification model yielded an AUROC of 0.92, 71% sensitivity, 85% specificity, and 84% accuracy.

At present, there is an elevated number of valuable techniques to detect liver fibrosis, but only a few are focused on HCV recurrence after liver transplantation^8, 10^. The diagnostic accuracy of the current model is excellent as compared to several tests aimed to predict development of severe recurrence after liver transplantation: ELF 0.827, IP-10 0.687, Fibroscan 0.760, APRI 0.833, and FIB-4 0.808 in 72 patients with HCV^7^. Additionally, the AUROC for the ELF test performed in 65 patients of the studied cohort (33 patients rapid fibrosers and 32 slow fibrosers) was 0.687, with an accuracy of 0.69 and a cut-off of 10.83. In addition, some imaging techniques have limited applicability in case of obesity and limited operator experience, such as transient elastography, Fibroscan^®^, which is considered when stratifying patients for antiviral treatment^7^. This technique made it possible to find two different patterns of fibrosis progression in patients with HCV one year after liver transplantation, with AUROCs of 0.92 and 0.80 in the estimation and in the validation groups, respectively^42^. Though, the number of patients included in the study was small (84 in total). In any case, each method targets different aspects of the same disease and then, the combination of some of these markers, occasionally, would improve the quality of the diagnosis.

Our metabolomics test was developed with serum from 203 patients from the Clínic Hospital in Barcelona (Spain). This approach is very powerful due to the strict follow-up of the volunteers of the study. All the patients were histologically diagnosed; and therefore, the study comprises the inherent limitations of liver biopsy. We have used the leave-one-out cross-validation (LOOCV) because it is an exhaustive method, *i.e*. it learns and tests on all possible ways to divide the original sample into a training and a validation set. LOOCV is a particular case of leave-p-out cross-validation with p = 1. Therefore, as it trains and tests on every point, LOOCV is as accurate to obtain approximations of generalization as splitting the original dataset into two-parts (training and testing), and using the testing score as a generalization measure^43, 44^. On the other hand, our test could be enhanced by including more patients from many other hospitals. Therefore, the accuracy of the method would allow for the more homogenous use of the discovered biomarkers and then, simplify their introduction into clinical practice.

We have observed a characteristic profile of the circulating levels of amino acid and lipid metabolites in all the METAVIR fibrosis stages in patients with HCV. The most relevant chemical groups distinguishing between rapid and slow fibrosers after liver transplantation were bile acids and sphingomyelins. Bile acids accumulate during cholestasis and may induce hepatocyte apoptosis^45^. However, each bile acid species may involve different biological effects. For example, taurochenodeoxycholic acid, which increased in the serum of rapid fibrosers, has been described as potential biomarker for liver cirrhosis in hepatitis B virus (HBV) patients^46^. This bile acid has been shown to activate PI3K-dependent survival pathways, which prevent their otherwise inherent toxicity and had antagonistic actions on pulmonary fibrosis in mice^47^. Some specific sphingomyelins rose very significantly along with liver fibrosis severity in the studied patients after liver transplantation. This result may support the proposed involvement of acidic sphingomyelinase, a known regulator of death receptor and stress-induced hepatocyte apoptosis, in liver fibrogenesis^48^. Indeed, sphingolipids are associated with liver fibrosis progression and poor treatment outcome in HCV^49^.

Lysophosphatidylcholines have been found dysregulated in association with increased mortality and severity of disease reflecting hepatocyte cell death in decompensated cirrhosis^34^. In our case, the levels of PC(20:4/0:0), PC(22:6/0:0), were increased in the serum of rapid fibrosers when compared with slow fibrosers; in contrast, the levels of PC(18:2/20:4), and PC(40:8) decreased. Also, these compounds were altered in the plasma of rats with induced fibrosis^50^. However, their levels decreased by chronic exposure to carbon tetrachloride and increased when exposed to lomustine. This difference may be due to many factors, such as the inherent lipid composition of each animal species and the fact that, depending on the insult, fibrosis may occur in different areas of the hepatic lobule shifting dissimilar metabolic pathways^51–54^.

In 2006, Zhang and colleagues described an algorithm to discriminate HCV patients with advanced liver fibrosis consisting on eight amino acids that reached an AUC of 0.92 ± 0.04^55^. This study only included 53 patients with fibrosis stages F3 and F4 *vs*. F0/F1-F2. In contrast, in our study, we differentiate between F0, F1 and F2-F4. On the other hand, the so-called Fischer-ratio^56^, BCAA/ArAA ratio and BTR ratio decreased in line with fibrosis severity in transplanted patients. It is well known that during hepatic failure, plasma levels of BCAA decrease and those of ArAA increase^56^. The BTR ratio has been described to be both a prognostic factor for early HCC and a predictive factor for recurrence^57^. According to this study, survival rates were significantly higher in patients with high baseline BTR. This affirmation is coherent with our results since the patients with advanced fibrosis after liver transplantation have smaller survival expectations and may develop HCC^58^.

Wondering if the signature found for the different fibrosis stages in patients with HCV after transplant could have some similarities to that of non-transplanted patients, we compared the presented results with those obtained in a similar study with 311 non-transplanted HCV patients (manuscript under preparation). Concretely, the levels of taurochenodeoxycholate, taurocholate, glycocholate, tyrosine and PC(16:0/16:0) phosphatidylcholine were found very significantly increased in F2-F4 patients when compared to F0 and F1 in the serum of both, transplanted and non-transplanted patients. From this study we can conclude that, although there are a number of similarities in the metabolic profiles of the different fibrosis stages, the multivariate biomarker present in the model for transplanted patients is so specific that it is not suitable for other pathologic states. All this new data on fibrosis and metabolomics will help to increase our understanding of the molecular events arisen and to develop novel non-invasive tests for other pathologies which involve fibrosis in their progression steps, such as HBV or NAFLD^20, 29^.

The current approach does not accomplish absolute quantification of the analytes due to causes inherent to the UHPLC-MS technique. As in our previous publications^29^ we did monitor the reproducibility of our assays, while taking steps to ensure that all data points included in the analysis were within the linear detection range of the platforms. Moreover, we previously confirmed that proton nuclear magnetic resonance (^1^H-NMR) is appropriate for depicting the metabolism of liver cirrhosis induced by HCV^19^. Yet, both techniques are complementary, *i.e*., UHPLC-MS is more sensitive and best suited for lipidomics analysis, while NMR is quantitative and focuses in central metabolism. It is our belief that the synergy of both strategies would improve the detection and absolute quantification of the found biomarkers.

## Conclusion

A distinctive metabolic fingerprint has been found in patients with different stages of fibrosis after liver transplantation. The diagnostic accuracy of this non-invasive technique is excellent compared with the most widely used and validated tests and open new possibilities to avoid invasive procedures. We are getting closer to a time when liver biopsy will be history.

### Study design

#### Patients

A total of 203 patients with HCV recurrence after LT were considered for the study in the Clínic Hospital, Barcelona, Spain. Exclusion criteria were: graft or patient survival shorter than 12 months after LT, combined kidney and LT, HBV or human immunodeficiency virus coinfection, presence of ascites, body mass index (BMI) over 33, chronic graft rejection, biliary tract complications, veno-occlusive disease, *de novo* autoimmune hepatitis, and recurrence of HCC during the first year after LT^42^. Patients were managed according to previously published protocols^42^. Liver biopsies and sera were collected; routine clinical analyses were performed. No patient received antiviral therapy at the moment of blood extraction. Most of patients with absent fibrosis did not receive relevant medication. The most common therapies in cirrhotic patients were diuretics, beta-blockers, or pain-killers.

One year after, LT patients were classified into five study groups using the METAVIR fibrosis staging system, which classifies liver fibrosis as absent (F0), restricted to the portal tract (F1), periportal or portal-portal septa with intact architecture (F2), bridging fibrosis with architectural distortion but no obvious cirrhosis (F3), and cirrhosis (F4)^59^. The minimal acceptable size of liver biopsy was considered 5 mm.

Patients were classified as “slow fibrosers” if they presented absent or minor fibrosis (fibrosis stages F0-F1) and as “rapid fibrosers” whether they presented fibrosis extending beyond the portal tracts (F2-F4). The study protocol was approved by the Investigation and Ethics Committee of the Hospital Clínic of Barcelona according to the ethical guidelines of the revised 1975 Declaration of Helsinki. Informed consent from all patients included in the study was obtained.

### Metabolomics analysis

#### Extraction of lipids and UHPLC-MS analysis

Serum samples were analysed following the procedure described by Barr *et al*.^29, 60^. Briefly, AA analysis was combined with two separate UHPLC-MS based platforms analysing methanol and chloroform/methanol serum extracts. Lipid and AA nomenclature follows the LIPID MAPS convention, http://www.lipidmaps.org/, and the Human Metabolome Database (HMDB), http://www.hmdb.ca, respectively.

For fatty acyls, BA, steroids and lysoglycerophospholipids profiling proteins were precipitated from 75 μl of defrosted serum samples by adding 300 μl of methanol in 1.5 ml microtubes at room temperature. The methanol used for extraction was spiked with metabolites not detected in unspiked human serum extracts (internal standards). After brief vortex mixing samples were incubated overnight at -20 °C. Supernatants were collected after centrifugation at 16,000 × g for 15 minutes, dried and reconstituted in methanol before being transferred to vials for UHPLC-MS analysis. Aliquots of 10 μl from the methanol extract were transferred to microtubes and derivatised for amino acid analysis^61^.

For glycerolipids, cholesteryl esters, sphingolipids and glycerophospholipids profiling, 10 μl of serum extracts were mixed with 10 μl of sodium chloride (50 mM) and 110 μl of chloroform / methanol (2:1) in 1.5 ml microtubes at room temperature. The extraction solvent was spiked with metabolites not detected in unspiked human serum extracts. After brief vortex mixing, samples were incubated for 1 hour at -20 °C. After centrifugation at 16,000 × *g* for 15 minutes, the organic phase was collected and the solvent removed. The dried extracts were then reconstituted in 100 μl of acetronitrile/isopropanol (1:1), centrifuged (16,000 × *g* for 5 minutes), and transferred to vials for UHPLC-MS analysis.

Randomized duplicate sample injections were performed, and, additionally, two different types of quality control (QC) samples were used to assess data quality^62^. QC are reference serum samples, which were evenly distributed over the batches and extracted and analysed at the same time as individual samples. QC calibration samples were used to correct the different response factors between and within batches and QC validation samples were used to assess how well data pre-processing procedure improved data quality^63^.

### Data pre-processing

In order to avoid systematic bias in the analysis, all samples were randomized *prior to* the metabolite extraction procedure and analysed blinded to the clinical data. Data pre-processing was performed following the procedure described before^60^. In brief, data obtained were pre-processed with the TargetLynx application manager for MassLynx (Waters Corp., Milford, MA). LC-MS features were identified prior to the analysis, either by comparison of their accurate mass spectra and chromatographic retention time (Rt) with those of available reference standards or, where these were not available, by accurate mass MS/MS fragment ion analysis. Intra and inter-batch normalization was based on multiple internal standards and pool calibration samples approach^63^.

Data pre-processing generated a list of chromatographic peak areas for the metabolites detected in each sample injection. An approximated linear detection range was defined for each identified metabolite, assuming similar detector response levels for all metabolites belonging to a given chemical class represented by a single standard compound.

### Statistical Analyses

Metabolite relative concentration values were balanced and trend corrected following the procedure described by Martínez-Arranz *et al*.^63^. Principal Components Analysis (PCA) (data not shown) and orthogonal projection to latent structures (OPLS) multivariate analyses were applied by exporting the normalised data to the SIMCA-P+ software package (version 14.1 Umetrics, Sweden).

To find a statistical model to differentiate between rapid (F2-F4) and slow fibrosers (F0-F1) a linear discriminant analysis (LDA) MASS library from R Software (R version 3.2.0; R Development Core Team, 2010; http://cran.r-project.org) was used and LOOCV was performed to confirm the model. Box-Cox transformations were applied to the biomarker metabolite levels for correcting non-normally distributed data and used to calculate the classification algorithm. The diagnostic accuracy of the model to identify patients with rapid or slow fibrosis progression one year after liver transplantation was assessed using the AUROC curve. The optimal score cut-off values were selected on the basis of sensitivity, specificity, positive predictive value, negative predictive value, and positive, and negative likelihood ratios to identify significant fibrosis. The optimum cut-off point for the estimation group (0.54) was defined as that at which average diagnostic accuracy was a maximum.

To define specific patterns associated with disease progression, patients histologically diagnosed with F0, F1, F2, F3, and F4 fibrosis stages were compared with one-way ANOVA and with Tukey *post-hoc* analysis. Univariate statistical analyses were also performed calculating group percentage changes and unpaired Student’s t-test p-value (or Welch’s t-test where unequal variances were found) for the comparison of rapid and slow fibrosers. In addition, Pearson correlation coefficient was calculated to detect the metabolites whose levels change in line with fibrosis progression. These analyses were performed using R with MASS, xlsx, robustbase and pwr packages.

Data are represented as mean ± SD unpaired Student’s t-test p-value (or Welch’s t-test where unequal variances were found). Significance was considered from a p-value < 0.05.

## Electronic supplementary material

Supplementary materials and methods

Dataset 1
