# Supplementary material for: A Metabolomics Signature Linked To Liver Fibrosis In The Serum Of Transplanted Hepatitis C Patients
Source: Sci Rep. 2017 Sep 5;7:10497. doi: 10.1038/s41598-017-10807-y (PMC5585246; doi:10.1038/s41598-017-10807-y)
Supplement: Supplementary file 1 — Supplementary materials and methods [file 41598_2017_10807_MOESM1_ESM.pdf]

A metabolomics signature linked to liver fibrosis in the serum of transplanted hepatitis C patients

Ainara Cano[1*], Zoe Mariño[2], Oscar Millet[3,4], Ibon Martínez-Arranz[1], Miquel Navasa[2], Juan Manuel Falcón-Pérez[3,4], Miriam Pérez-Cormenzana[1], Joan Caballería[2], Nieves Embade[3], Xavier Forns[2], Jaume Bosch[2], Azucena Castro[1], José María Mato[3]

[1]OWL, Parque Tecnológico de Bizkaia, Derio 48160, Bizkaia, Spain

[2]Liver Unit, Hospital Clínic, Centro de Investigación Biomédica en Red de Enfermedades Hepáticas y Digestivas (CIBERehd); Institut d'Investigacions Biomediques August Pi Sunyer (IDIBAPS), Barcelona, Spain.

[3]Metabolomic Unit, CIC bioGUNE, CIBERehd, Parque Tecnológico de Bizkaia, Derio 48160, Spain.

[4]Ikerbasque, Basque Foundation for Science, Bilbao, Bizkaia, Spain

*****Corresponding author

Supplementary materials and methods

UHPLC-MS Analysis

Targeted serum metabolic profiles were semiquantified as previously described. An ultra-high performance liquid chromatography (UHPLC) single quadrupole-mass spectrometry (MS) amino acid analysis system was combined with two separate UHPLC time-of-flight MS based platforms analysing methanol and chloroform/methanol serum extracts. Chromatographic separation and mass spectrometric detection conditions for each platform are summarized in Supplementary Table S1.

Although there is no single method to analyse the entire lipidome of a biological sample, mainly due to the wide concentration range of the metabolites and their extensive chemical diversity, the current study has been designed for an extensive coverage of lipids and amino acids. Identified ion features in the methanol extract platform included non-esterified fatty acids, acyl carnitines, N-acyl ethanolamines, bile acids, steroids, oxidized fatty acids, monoacylglycerophospholipids, and monoetherglycerophospholipids. The chloroform/methanol extract platform covered glycerolipids, cholesteryl esters, sphingolipids, diacylglycerophospholipids, acyl-etherglycerophospholipids and primary fatty acid amides. Each extract was spiked with

metabolites not detected in unspiked human serum extracts: tryptophan-d5 (indole-d5), PC(13:0/0:0), NEFA(19:0) and dehydrocholic acid in methanol extract; SM(d18:1/6:0), PE(17:0/17:0), PC(19:0/19:0), TAG(13:0/13:0/13:0), TAG(17:0/17:0/17:0), Cer(d18:1/17:0) and ChoE(12:0) in chloroform/ methanol extract.

Supplementary Table S1. UHPLC-MS analysis methods

	**Platform 1**	**Platform 2**	**Platform 3**
**Column type**	UPLC BEH C18, 1.0 x 100 mm, 1.7 µm	UPLC BEH C18, 2.1 x 100 mm, 1.7 µm	UPLC BEH C18, 1.0 x 100 mm, 1.7 µm
**Flow rate**	0.14 ml/min	0.40 ml/min	0.14 ml/min
**Solvent A**	H_2O + 0.05% Formic Acid	H_2O + ACN + 10mM Ammonium Formate	10mM Ammonium Bicarbonate (pH = 8.8)
**Solvent B**	ACN + 0.05% Formic Acid	ACN+ Isopropanol + 10mM Ammonium Formate	ACN
**(%B), time**	0%, 0 min	40%, 0 min	2%, 0 min
**(%B), time**	50%, 2 min	100%, 10 min	8%, 6.5 min
**(%B), time**	100%, 13 min	40%, 15 min	20%, 10 min
**(%B), time**	0%, 18 min	40%, 17 min	30%, 11 min
**(%B), time**	-	-	100%, 12 min
**(%B), time**	-	-	2%, 14 min
**Column temperature**	40 °C	60 °C	40 °C
**Injection volume**	2 µl	3 µl	2 µl
**Source temperature**	120 °C	120 °C	120 °C
**Nebulisation N_2 flow**	600 l / hour	1000 l / hour	600 l / hour
**Nebulisation N_2 temperature**	350 °C	500 °C	350 °C
**Cone N_2 flow**	30 l / hour	30 l / hour	10 l / hour
**Capillary voltage**	2.8 kV	3.2 kV	3.2 kV
**Cone voltage**	50 V	30 V	30 V

Metabolite Identification

The LC–MS features (as defined by retention time, mass-to-charge ratio pairs, Rt-m/z), were identified prior to the analysis, either by comparison of their accurate mass spectra and chromatographic Rt with those of available reference standards or, where these were not available, by accurate mass Online tandem mass spectrometry (MS/MS) fragment ion analysis. MS/MS experiments for metabolite identification were performed on a Waters QTOF Premier (Waters Corp.) and a Waters SYNAPT G2 instrument, operating in both the positive and negative ion electrospray modes.

LC−MS features were associated with non-esterified fatty acids NEFA, bile acids, oxidized fatty acids, free sphingoid bases, and amino acids by comparison of their accurate mass spectra and chromatographic retention times in commercial serum metabolite extracts (PromoCell Inc., Germany) with those obtained using available reference standards (mass fragment accuracy was <3 ppm for m/z 400−1000, and <1.2 mDa for m/z 50−400).

For all other species (acyl carnitines, diacylglycerols, triacylglycerols, cholesterol esters, glycerophosphocholine, glycerophosphoethanolamine, glycerophosphoinositol, sphingomyelin, ceramides, and monohexosylceramides) a theoretical m/z database was first generated for all possible combinations of fatty acid derived moieties. The association of detected Rt-m/z pairs with lipid species contained in the theoretical database was subsequently established either by comparison of their accurate mass spectra and chromatographic Rt with those obtained using available reference standards or, where these were not available, by accurate mass MS/MS fragment ion analysis (mass fragment accuracy was <3 ppm for m/z 400−1000, and <1.2 mDa for m/z 50−400).

Supplementary Legends

Supplementary Table S1. Individual composition of lipid and amino acid species in the serum of HCV patients with liver fibrosis. Features of the analysed metabolites obtained through OPLS multivariate analysis (A), ANOVA´s Tukey post-hoc and Pearson analyses (B), and unpaired t test (C). The relative metabolite levels in serum samples of each fibrosis stage have been compared with its immediate lower fibrosis degree in the ANOVA and Tukey post-hoc analyses. Rapid (F2-F4) and slow (F0-F1) fibrosers have been compared with unpaired Student’s t test (or Welch´s t test). The nomenclature for the lipid species follows these rules: glycerolipids and

ChoE species: C:D, where C is the number of carbon atoms and D is the number of double bonds. The position of the double bonds in the acyl chains is not considered, as well as the position of the acyl chains. Sphingolipids species: dA:B/C:D, where dA:B represents the sphingoid base d18:1, sphingosine; d18:2, sphingadiene; d18:0, sphinganine and C:D indicates the number of carbon atoms C, and double bonds D, contained in the N-linked fatty acid. Glycerophospholipids as diacyl, monoacyl, monoether or monoether-monoacyl species: A:B/C:D, where A:B and C:D refer to the number of carbon atoms and number of double bonds contained in the sn-1 and sn-2 side chains, respectively. For glycerophospholipids containing an ether moiety the prefix, O-, denotes the presence of an alkyl ether (plasmanyl) substituent, whereas the prefix P- refers to a vinyl ether (alkenyl or plasmenyl) substituent. The suffix, e, indicates the presence of an ether linked substituent, although plasmanyl or plasmenyl classification has not been confirmed. X:Y nomenclature (where X = A+C and Y = B+D) is used where evidence was found for the contribution of multiple species to a single chromatographic peak. Overlapping of two or more metabolites or non-confirmed identification is indicated in “Individual composition or probable ID”. An “x” in the name of some NEFA, FAA and NAE indicates the unknown position of the double bounds. Most of the oxidised fatty acids are not identified, but sub-classified as hydroxy-eicosatetraenoic acids, epoxy-eicosatetraenoic acids, hydroxy-octadecadenoic acids, oxo-octadecadenoic acids or dihydroxyeicosatrienoic acids.

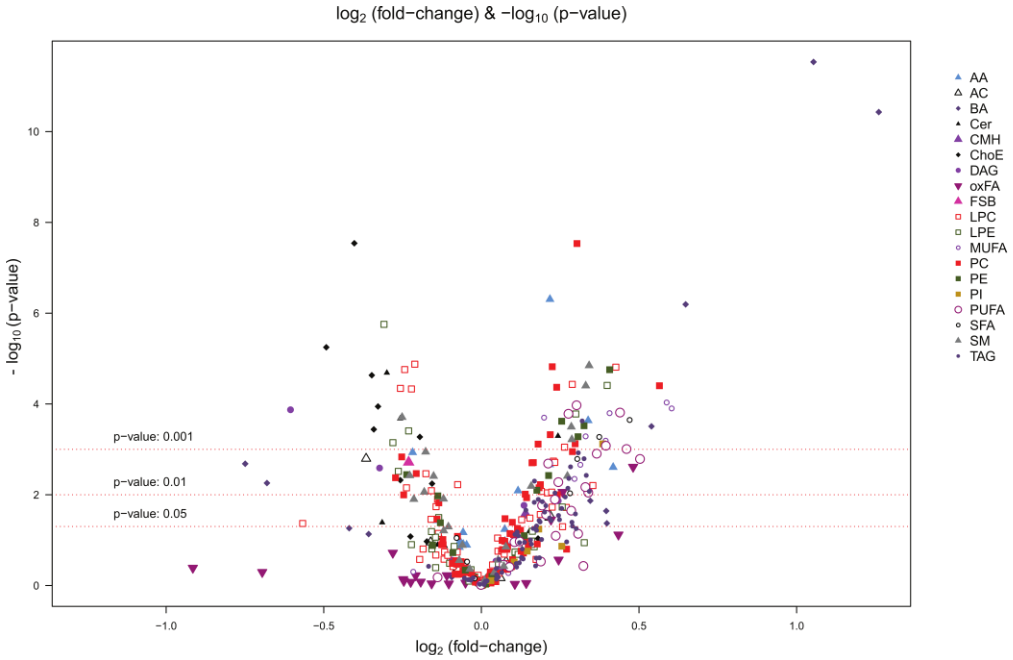

Supplementary Figure S1. Volcano plot [log10(p-value) *vs*. log2(fold-change)] for the comparison of rapid fibrosers [F2-F4 (n=69)] *vs*. slow fibrosers [F0 and F1 (n=134)] one year after liver transplantation. The volcano plot highlights the most significant lipids and amino acids considered individually. Regarding their p-value, the most altered lipid levels were taurochenodeoxycholic acid (TCDCA), taurocholic acid (TCA), and glycocholic acid (CGA), ChoE(18:1), PC(16:0/16:0), and tyrosine. Amino acids (AA), Acyl carnitines (AC), bile acids (BA), ceramides (Cer), monohexosylceramides (CMH), cholesteryl esters (ChoE), diacylglycerols (DAG), oxidized fatty acids (oxFA), free sphingoid bases (FSB), lysophosphatidylcholines (LPC), lysophosphatidylethanolamines (LPE), monounsaturated fatty acids (MUFA), phosphatidylcholines (PC), phosphatidylethanolamines (PE), phosphatidylinositols (PI), polyunsaturated fatty acids (PUFA), saturated fatty acids (SFA), sphingomyelins (SM), triacylglycerols (TAG).
